# The impact of deprivation on colorectal cancer‐stage distribution in a setting with high hospital bed density: A Japanese multilevel study

**DOI:** 10.1002/cam4.70042

**Published:** 2024-07-24

**Authors:** Toshiaki Shibata, Daisuke Shinjo, Kiyohide Fushimi

**Affiliations:** ^1^ Department of Health Policy and Informatics Tokyo Medical and Dental University Graduate School Bunkyo‐ku Tokyo Japan

**Keywords:** deprivation, early detection, health equality, healthcare access, stage at diagnosis

## Abstract

**Background:**

A methodology for determining the appropriate balance between medical access and combating poverty remains undetermined. To address the boundary conditions for exceedingly good medical access, this study examined whether the impact of deprivation on cancer stage distribution could be eliminated in Japan, which has the highest hospital bed density in the world.

**Methods:**

A nationwide medical claims‐based database was used to evaluate the influence of municipality‐level hospital bed density and the postal code‐level areal deprivation index on cancer stage at diagnosis. Given the limited number of similar studies in Japan, we focused on colorectal cancer (CRC), for which disparities have been reported in a prefecture‐level study. Multilevel multivariate logistic regression models were used, with odds ratios (ORs) and 95% confidence intervals (CIs) adjusted for baseline and socioeconomic factors.

**Results:**

Regardless of the early/advanced‐stage definitions, CRC consistently tended to be detected at more advanced stages in more deprived areas. In the analysis of stages 0–I/II–IV, the OR (95% CI) was 1.09 (1.05, 1.14) (*p* < 0.001). In the analyses of stages 0–I/II–IV and 0–II/III–IV, gradients were observed, and later detections were observed for more deprived segments. Hospital bed density was not significantly associated with the stage distribution.

**Conclusion:**

The results indicate that inequalities in CRC detection due to deprivation persist even in the country with the highest hospital bed density worldwide, suggesting that poverty measures remain indispensable regardless of hospital bed access. Further investigation of various regions and cancers is required to develop a practical framework.

## Graphical abstract

The study findings suggest that the impact of socioeconomic deprivation on colorectal cancer stages at diagnosis persists even with exceptionally high hospital bed density. To ensure equal access to healthcare, public health administrators should promote not only medical access but also measures to tackle poverty.

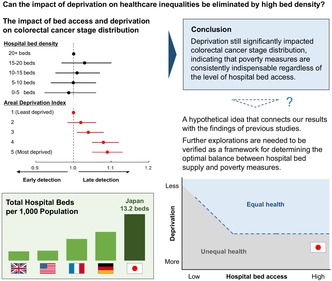

## 1 INTRODUCTION

Previous studies have shown that socioeconomic deprivation (SED) affects various cancer care indicators such as stage at diagnosis and mortality rate.^1^, ^2^ The impact of SED on health varies depending on the medical system and resources of each region^3^; hence, a comprehensive framework is required to determine necessary social interventions in a given regional medical setting. However, while there are some frameworks to identify social determinants of health,^4^, ^5^ to our knowledge, an intervention‐focused framework remains undetermined. To obtain such a framework, it is necessary to consider the impact of SED on various medical systems and resource environments, which requires further research. Thus, this study focused on investigating how SED affects the status at diagnosis of cancer patients under a universal health system.

In this context, it is meaningful to select Japan, which has an extremely high hospital bed density and public health insurance coverage. According to the Organization for Economic Co‐operation and Development Health Statistics 2023, Japan has the largest number of hospital beds per capita worldwide (13.2 hospital beds per 1000 people, 2018), which is the effective upper limit compared with the United States (2.8 beds), United Kingdom (2.6 beds), France (6.1 beds), and Germany (8.1 beds).^6^ In addition, the physician density per 1000 people (2018) in Japan (2.5) is comparable with that in the United States (2.6), the United Kingdom (2.8), and France (3.1).^6^ Therefore, if disparities in cancer stage distribution due to SED are observed in Japan, the influence of SED may not be fully eliminated, even if abundant medical access is ensured.

Additionally, this study focused on colorectal cancer (CRC), the most common (2019)^7^ and the second deadliest (2021)^8^ cancer in Japan. This is because one of the few studies in Japan focusing on the impact of SED on cancer stage distribution,^9^, ^10^ the study by Ito in Osaka (2017), explored the impact of areal deprivation on stage distributions of gastric, colorectal, lung, breast, prostate, and cervical cancer and revealed that, among cancers common to men and women, the influence of SED on early‐stage detection was significantly growing, particularly for CRC.^11^

It is useful to use the cancer stage at diagnosis as an outcome as it can be considered both a major milestone for achieving good cancer treatment outcomes^12^ and a good indicator of the impact of social determinants of health, independently of care quality. Numerous studies have been conducted on the influence of SED on the CRC stage at diagnosis, demonstrating that more severe poverty leads to more advanced cancers at diagnosis.^13^, ^14^ Among these, we focused on studies that investigated the impact of SED and medical access on CRC stage distribution in universal health systems. The association between advanced CRC stages and deprived areas in the United Kingdom,^15^ inner and outer regional areas (i.e., outside of major cities) in Australia,^16^ and residences' distance from the nearest cancer centers (≥58 km) in Scotland^17^ have been reported. These previous studies did not involve countries with high bed densities, and the impact appeared to be greater when bed access was very low. In light of this, if the impact could be investigated when the bed density is very high, we would be able to obtain clearer perspectives on a unified mapping of the combined impacts of SED and bed access on the CRC stage at diagnosis.

This study aimed to examine whether SED affected CRC stages at diagnosis under conditions of exceedingly high hospital bed density. To the best of our knowledge, this is the first study to investigate the extent to which SED in a country with the highest hospital bed density worldwide affects the stage at diagnosis, using a nationwide database. Furthermore, this study is the first of its kind to propose a possibility for building a comprehensive framework that considers the optimal combination of hospital bed supply and poverty measures to achieve equality in medical access.

## 2 MATERIALS AND METHODS

### 2.1 Study design

This retrospective cross‐sectional study was based on the Diagnosis Procedure Combination/Per‐Diem Payment System (DPC/PDPS) database,^18^ a nationwide medical claims‐based database for acute inpatients comprising information regarding claims for reimbursements as well as patient information, such as cancer stage at diagnosis, insurance type, and postal code of residence. The areal deprivation indicator (ADI)^19^ at the postal code level was used as an indicator of poverty. By analyzing these data, the influence of ADI on CRC stages at diagnosis was assessed. In our model, if ADI was significantly associated with the later detection of CRC, we concluded that SED was not fully normalized, even by an exceedingly high hospital bed density. Since this study focused on inpatients (48.6% of CRC treatments among DPC member hospitals^20^), on whom the impact of SED was expected to be smaller than on outpatients, the results can provide observations under more conservative conditions.

### 2.2 Study population

**FIGURE 1:**
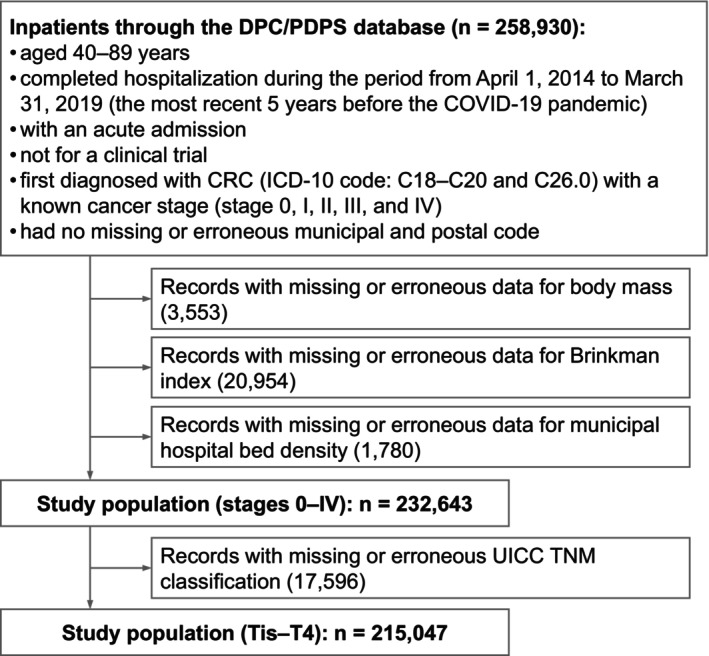
Inclusion and exclusion criteria.

Herein, anonymized data extracted from the DPC/PDPS database were used, which contained information from both DPC member hospitals (1165), providing acute care, and nonmember hospitals (98), in combination with the Census^21^, ^22^ and municipal statistics databases.^23^ The patients included in the study were (1) aged 40–89 years; (2) admitted to and discharged from hospitals between April 2014 and March 2019; (3) hospitalized in the acute phase regardless of their admission route for newly diagnosed CRC (C18.0–C18.9 and C26.0 for colon cancer and C19.9 and C20.9 for rectal cancer^24^) as the disease that required the most medical resources; (4) not hospitalized for a clinical trial (the data of stage at diagnosis may be the cancer stage when rediagnosed at the clinical trial admission); and (5) without missing or erroneous postal and municipal codes. The exclusion criteria were as follows: (1) missing data for body mass index (BMI) and Brinkman index (BI), and (2) hospital bed density. Besides, for the analysis of Tis–T1 versus T2–4, (3) records with missing TNM classification data^25^ were also excluded. Consequently, 232,643 inpatients (1794 municipalities, 53,564 postal code areas) were identified for the main analyses (Figure 1). The difference in the percentage of patients with stages 0–I cancer between the study and excluded populations was 0.8%.

### 2.3 Primary outcome

The primary outcome was the detection of CRC at stages 0–1, for which the 5‐year survival rate is over 90%, and standard treatments are endoscopic surgeries,^26^ which are less invasive and less costly.^27^, ^28^ In addition, to broadly assess the impact of SED, a range of early‐stage definitions, including stages 0–II and Tis–T1, were explored. Cancer staging was performed according to the Japanese Classification of Colorectal, Appendiceal, and Anal Carcinoma,^29^ consistent with the Union Internationale Contre le Cancer staging system.^25^

### 2.4 Explanatory variables

ADI was used as an explanatory variable. ADI is a composite measure of census variables designed to summarize SED. The Japanese version of ADI was developed by Nakaya.^10^ Herein, the census data^22^ was reaggregated to obtain postal code‐level ADIs. Furthermore, the census database was integrated with the postal code database by matching place names. The place names for each postal code were extracted from zipcloud,^30^ a commercial database cleansing the data provided by the Japan Post,^31^ and the notations of place names were aligned by establishing the notation rules. The population coverage rate of the matched areas was 98.4% of Japan's total population.

### 2.5 Independent variables

Sex, age group, cancer site, BMI (categorized by <20.0, 20.0–24.9, ≥24.9 kg/m^2^ for 40–64 years; <21.5, 21.5–24.9, ≥24.9 for 65–89 years),^32^, ^33^ BI,^34^ and Charlson comorbidity index^35^, ^36^ were used as baseline factors. As an individual socioeconomic factor, individual income levels, available for patients aged ≥70 years in the DPC/PDPS database (dichotomized by whether working‐age‐level or not), were used. Furthermore, municipality‐level hospital bed density^23^ was used to adjust for municipal medical access. We used the number of beds obtained by subtracting psychiatric beds, infectious disease beds, tuberculosis beds, and recuperation beds from the total number of hospital beds nationwide to indicate the number of acute care beds for noncommunicable physical diseases. Additionally, because the meaning of municipal hospital bed density can vary depending on the major transportation mode in those regions, the postal code‐level transportation mode (percentage of people commuting by car or motorcycle, categorized in 20% increments)^21^ was taken into consideration.

### 2.6 Statistical analyses

Because our analyses encompassed three layers—patient, postal code area, and municipality—multilevel multivariate logistic regression models were employed. An odds ratio (OR) <1 indicated the detection of earlier stages of CRC. Odds ratios (ORs) and 95% confidence intervals (CIs) were adjusted for individual baseline factors and socioeconomic indicators.

The need for subgroup analyses to identify vulnerable social groups was examined by assessing the impact of the interaction terms between the highest ADI (most deprived) and individual/areal attributes on the CRC stage distribution.

Validation of ADI was carried out through two methods: (1) comparison of ADI distribution statistics with those in a previous study using the DPC/PDPS database, and (2) comparison with the distribution of individual income level of those aged ≥70 years. The representativeness of the study population was evaluated by comparing the CRC stage distribution with that of a previous Japanese study.

To analyze sensitivity, the robustness of the model was assessed by adjusting for the following municipal‐level indicators: (1) population size and (2) gastroenterologist density (the per‐capita number of doctors who designate gastroenterology as their medical specialty at a hospital or clinic). In addition, since the analyses using the DPC/PDPS database can overestimate the number of advanced‐stage cases as it is based on institutional patient IDs and patients who received continued treatment across hospitals can be misperceived as new patients, the model was tested by excluding stage IV and stages III–IV. Additionally, regional differences in stage distribution in terms of municipal population size and hospital bed density were investigated. Furthermore, to assess the impact of the lack of granularity in areal‐level ADI, we conducted an analysis using individual income level instead of ADI for patients aged ≥70 years, for whom the individual‐level income category is available. Statistical analyses were conducted using R ver. 4.0.2 and EZR ver. 1.54.^37^

## 3 RESULTS

### 3.1 Validation of ADI

The ADI distribution was compared with that in the study by Tomioka.^38^ Our ADI statistics (mean, median, maximum, and minimum) based on the Census 2015 were 0.00, −0.096, 14.5, and −5.21, respectively, whereas those in Tomioka's study based on the Census 2010 were 0.00, −0.08, 10.36, and − 4.03, respectively. Additionally, in comparison with the distribution of individual income levels for those aged ≥70 years (Table S1), a gradient was observed in which the percentages of higher‐income patients were smaller in areas with higher ADIs.

### 3.2 Patient demographics

A chi‐squared test showed that the more deprived regions tended to have a lower rate of early CRC detection (Table 1). A gradient was observed, in which the likelihood of late CRC detection (stages II–IV) was associated with higher ADIs. A gradient was also observed between areas with higher percentages of people commuting by car or motorcycle and late CRC detection.

**TABLE 1:** Patient demographics by CRC stage distribution (Stages 0–I vs. II–IV).

	Stages 0–I vs. II–IV (*N* = 258,930)					
	Total	Early *n* (%)		Advanced *n* (%)		*p* *
Age group						
40–64 years old	73,565	25,285	(34.4)	48,280	(65.6)	<0.01
65–89 years old	185,365	59,647	(32.2)	125,718	(67.8)	
Sex						
Female	109,734	34,866	(31.8)	74,868	(68.2)	<0.01
Male	149,196	50,066	(33.6)	99,130	(66.4)	
Cancer site						
Rectum	95,877	30,390	(31.7)	65,487	(68.3)	<0.01
Colon	163,053	54,542	(33.5)	108,511	(66.5)	
BMI						
<Standard range	89,770	24,280	(27.0)	65,490	(73.0)	<0.01
Within std. range	105,129	36,756	(35.0)	68,373	(65.0)	
≥Standard range	60,478	23,028	(38.1)	37,450	(61.9)	
BI						
<800	199,965	66,091	(33.1)	133,874	(66.9)	<0.01
≥800	37,176	11,622	(31.3)	25,554	(68.7)	
CCI						
<3	210,581	79,542	(37.8)	131,039	(62.2)	<0.01
≥3	48,349	5390	(11.1)	42,959	(88.9)	
Municipal hospital bed density (per 1000 population)						
≥20 beds	27,130	8626	(31.8)	18,504	(68.2)	<0.01
15–20	46,062	14,988	(32.5)	31,074	(67.5)	
10–15	74,267	24,021	(32.3)	50,246	(67.7)	
5–10	79,714	26,678	(33.5)	53,036	(66.5)	
0–5	29,861	10,038	(33.6)	19,823	(66.4)	
Municipal gastroenterologist density(per 1000 population)						
≥3	29,801	9867	(33.1)	19,934	(66.9)	<0.01
2–3	35,319	11,923	(33.8)	23,396	(66.2)	
1–2	78,442	25,735	(32.8)	52,707	(67.2)	
0.5–1	64,783	21,152	(32.7)	43,631	(67.3)	
0–0.5	48,689	15,674	(32.2)	33,015	(67.8)	
Transportation mode (postal code‐level rate of people communing by car or motorcycle)						
0%–20%	35,860	12,427	(34.7)	23,433	(65.3)	<0.01
20%–40%	50,937	17,323	(34.0)	33,614	(66.0)	
40%–60%	36,079	11,866	(32.9)	24,213	(67.1)	
60%–80%	71,116	22,743	(32.0)	48,373	(68.0)	
80%–100%	63,042	19,992	(31.7)	43,050	(68.3)	
Municipal population						
500 k+	19,004	6350	(33.4)	12,654	(66.6)	<0.01
300–500 k	40,211	13,339	(33.2)	26,872	(66.8)	
100–300 k	121,294	39,969	(33.0)	81,325	(67.0)	
50‐100 k	40,198	12,769	(31.8)	27,429	(68.2)	
0–50 k	38,223	12,505	(32.7)	25,718	(67.3)	
ADI (postal code‐level)						
1 (Least deprived)	27,633	9571	(34.6)	18,062	(65.4)	<0.01
2	53,677	18,263	(34.0)	35,414	(66.0)	
3	63,697	21,131	(33.2)	42,566	(66.8)	
4	62,657	19,902	(31.8)	42,755	(68.2)	
5 (Most deprived)	51,266	16,065	(31.3)	35,201	(68.7)	

Abbreviations: ADI Areal Deprivation Index; BI, Brinkman Index; BMI, Body mass index; CCI, Charlson Comorbidity Index; CRC, colorectal cancer.

* Chi‐square test.

### 3.3 Multilevel multivariate logistic regression model

**FIGURE 2:**
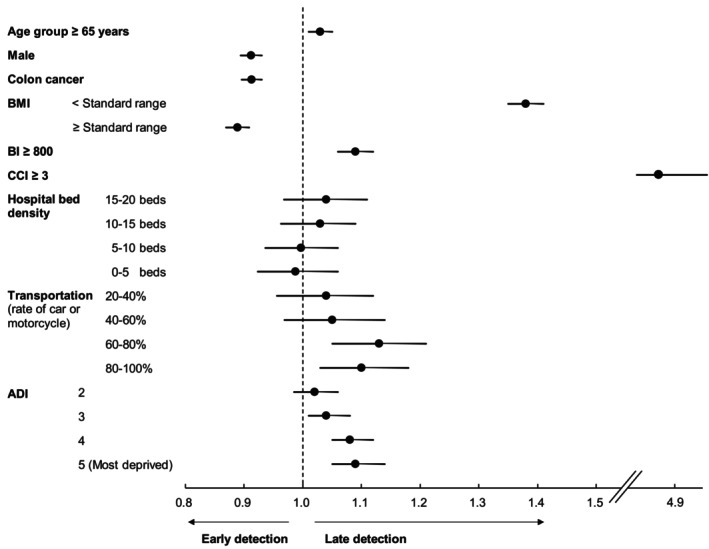
ORs for advanced CRC stage (Stages 0–I vs. II–IV). A gradient was observed in which more deprived populations were more likely to be diagnosed at stages II–IV. CRC, colorectal cancer; OR, odds ratio.

Multilevel multivariate logistic analyses of stages 0–I versus II–IV (Table 2, Figure 2) showed that when the ADI quintile was 5 (most deprived), 4, and 3, the ORs (95% CIs) were 1.09 (95% CI: 1.05, 1.14), 1.08 (95% CI: 1.05, 1.12), and 1.04 (95% CI: 1.01, 1.08), respectively. Similar tendencies were consistently observed in the analysis of stages 0–II versus III–IV and Tis–T1 versus T2–4 (Table S2, Figures S1 and S2). Municipal hospital bed density was not significantly associated with advanced CRC stages. Higher percentages of the population commuting by car or motorcycle were associated with advanced CRC stages, regardless of the definition of early or advanced stages. ORs of the higher age group for advanced CRC stages varied according to the early/advanced stage definitions; for the analysis that defined early stage as stages 0–I and Tis–T1, ORs were >1, while for the analysis that defined early stage as stages 0–II, ORs were <1. The variance inflation factors for the variables in this model were consistently <2.0. No significant association was observed between ADI and hospital bed density (*R* ^2^ = 0.025).

**TABLE 2:** Multilevel multivariate logistics regression for advanced CRC stage (Stages 0–I vs. II–IV).

	OR for advanced CRC stage, Stages 0–I vs. II–IVs		
	OR	95% CI	*p*
Age group			
≤64 years [ref.]			
≥65 years	1.03*	[1.01–1.05]	0.011
Sex			
Male	0.91***	[0.89–0.93]	<0.001
Female [ref.]			
Cancer site			
Colon	0.91***	[0.90–0.93]	<0.001
Rectum [ref.]			
BMI			
<21.5	1.38***	[1.35–1.41]	<0.001
21.5–24.9 [ref.]			
≥24.9	0.89***	[0.87–0.91]	<0.001
BI			
<800 [ref.]			
≥800	1.09***	[1.06–1.12]	<0.001
CCI			
<3 [ref.]			
≥3	4.85***	[4.70–5.01]	<0.001
Municipal hospital bed density (per 1000 pop)			
≥20 beds [ref.]			
15–20	1.04	[0.97–1.11]	0.295
10–15	1.03	[0.96–1.09]	0.433
5–10	1.00	[0.94–1.06]	0.932
0–5	0.99	[0.92–1.06]	0.718
Transportation mode (postal code‐level rate of people communing by car or motorcycle)			
0%–20% [ref.]			
20%–40%	1.04	[0.96–1.12]	0.392
40%–60%	1.05	[0.97–1.14]	0.230
60%–80%	1.13**	[1.05–1.21]	0.001
80%–100%	1.10**	[1.03–1.18]	0.005
ADI (postal code‐level)			
1 (Least deprived) [ref.]			
2	1.02	[0.99–1.06]	0.256
3	1.04*	[1.01–1.08]	0.022
4	1.08***	[1.05–1.12]	<0.001
5 (Most deprived)	1.09***	[1.05–1.14]	<0.001

Abbreviations: ADI, Areal deprivation index; BMI, Body mass index; BI, Brinkman index; CI, confidence interval; CCI, Charlson Comorbidity Index; CRC, colorectal cancer; OR, Odds ratio.

* *p* < 0.05.

** *p* < 0.01.

*** *p* < 0.001.

### 3.4 Subcategory analyses

No significant associations with CRC stage distribution were observed for combinations of the highest ADI and (1) population aged <65 years, (2) female sex, (3) colon cancer, and (4) areas with the lowest hospital bed densities (data not shown).

### 3.5 Sensitivity analyses

The statistical model was adjusted for municipal population size and gastroenterologist density, which resulted in the same tendencies regarding the influence of the ADI on CRC stage distribution (Table S3). The model without stage IV and stages III–IV cases showed a significant association between advanced stages and higher ADIs (Table S4). Similar results were obtained for the analysis using individual income level instead of areal‐level ADI for patients aged ≥70. In addition, regional fluctuations in the proportion of advanced stages were within 2.1%, and no consistent gradients were observed (data not shown).

## 4 DISCUSSION

This study explored the impact of SED on CRC stage distribution to clarify whether disparities remain even with Japan's exceedingly high bed density. The results showed that advanced CRC stages (stage II–IV, stage III–IV, and T2–4) are more likely to be detected in deprived populations, suggesting that even exceedingly high hospital bed density cannot fully eliminate health disparities due to SED. Therefore, for policymakers to ensure equality in healthcare, measures to combat poverty should always be considered regardless of hospital bed‐access levels. ADI‐stratified demographics (Table 3) represent that the most deprived patients were more likely to be observed in the depopulated areas (designated by the government^39^).

**TABLE 3:** ADI‐stratified demographics for CRC patients.

	ADI										*p* *
	1 (Least deprived)		2		3		4		5 (Most deprived)		
Total	27,633	(10.7%)	53,677	(20.7%)	63,697	(24.6%)	62,657	(24.2%)	51,266	(19.8%)	
Depopulated area											
Yes	726	(6.7%)	1177	(10.8%)	1607	(14.7%)	3042	(27.9%)	4359	(40.0%)	<0.01
No	26,907	(10.8%)	52,500	(21.2%)	62,090	(25.0%)	59,615	(24.0%)	46,907	(18.9%)	

Abbreviations: ADI, Areal Deprivation Index; CRC, Colorectal cancer.

* Chi‐squared test.

In contrast, variations in hospital bed density did not significantly affect the CRC stage distribution, implying that the impact of high hospital bed density may saturate or disappear above certain thresholds. If this is the case, the optimum hospital bed density may be lower than that in Japan (13.2 beds per 1000 population^6^).

**FIGURE 3:**
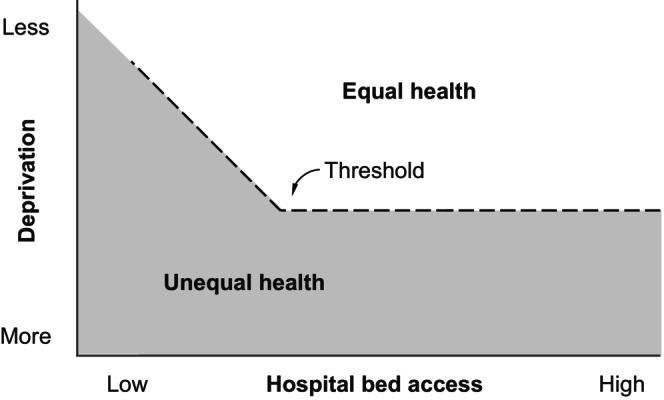
Hypothesis for combined impacts of SED and bed access on the CRC stage at diagnosis. This diagram is proposed as a new hypothetical idea that connects with the key findings of previous relevant studies. CRC, colorectal cancer; SED, socioeconomic deprivation.

Combining these aforementioned findings with the results of previous studies, it may be possible to create a hypothetical mapping as shown in Figure 3. In previous studies, bed access appeared to affect the CRC stage at diagnosis only in situations where bed access was extremely low. This study suggests that bed access does not affect the CRC stage at diagnosis in situations where hospital bed density is extremely high. Further research would allow this hypothetical mapping to be verified as a framework.

Intriguingly, our results also showed that more advanced CRC stages were detected in areas where people traveled mainly by cars or motorcycles. While numerous studies have reported the negative impact of car/motorcycle‐centered transportation modes on people's health,^40^, ^41^ to our knowledge, the impact of transportation modes on cancer stage distribution has rarely been examined. Further investigations need to be done to examine the underlying mechanisms.

The fact that patients aged ≥65 years tended to be diagnosed with stages 0–II cancer but not with 0–I or Tis–T1 cancer can be attributed to the difference in cancer screening behavior between generations. While older populations (65–79 years) rely more on municipal‐run CRC screening (55%)^42^ that utilizes less sensitive stool testing,^43^ the majority of younger populations (40–64 years) mainly undergo employment‐based CRC screening (63%)^42^ in which colonoscopy can be performed upon request.

Japan has a well‐established healthcare system that provides good medical access with universal public insurance, free‐of‐charge municipal CRC screening, and financial support for monthly co‐payments.^44^ Thus, to reduce health disparities, key issues may lie predominantly on the demand side, such as health literacy shortages or poor public transportations.^45^ Therefore, it is crucial for public health administrators to carefully identify practical and addressable factors that can influence people's behaviors.

Regarding CRC stage distribution, the percentage of stages 0–II cases in this study was 56.2% (40–89 years), whereas that in Toyoda's study in Osaka was 62.6% (≥50 years, 2012–2014),^46^ showing roughly consistent findings, considering the differences of regions, data periods, and age groups. Concerning the relationship between sex and CRC stage at diagnosis, our findings of men being more likely to be diagnosed at earlier stages are consistent with Toyoda's findings.^46^ Additionally, our observation of higher SED being associated with advanced CRC detection is consistent with those of Crawford and Baade.^15^, ^16^ Earlier detection of colon cancer contradicted previous studies in Switzerland^13^ and Australia^16^; however, this discrepancy could be explained by Japan's higher screening rate (41.4%, 2016)^42^ than in Switzerland (22.2%, 2012)^47^ or Australia (33.0%, 2013),^48^ which contributes to the detection of less symptomatic colon cancer.

This study has several limitations. First, this was a cross‐sectional study. Second, some CRC risk factors were not included in the model. Data regarding diet, alcohol consumption, genetic polymorphisms, family history, history of medical tests, and cancer screenings^49^ were not available in the DPC/PDPS database. Third, our analyses using the DPC/PDPS database may have overestimated the impact of advanced stages. However, similar trends were observed in the sensitivity analyses that excluded stage IV and stages III–IV cases. Fourth, because variables at the municipal level were used, various confounders can remain in our models. However, the models were robust when adjusted for municipal population size and gastroenterologist density. Finally, medical access was represented only by bed density. The distance to hospitals was considered in part through adjustments by transportation mode.

In conclusion, the study findings suggest that the impact of SED on CRC stages at diagnosis persists even with exceptionally high hospital bed density. To ensure equal access to healthcare, public health administrators should promote not only medical access but also measures to tackle poverty. In contrast, the fact that no significant associations were observed between hospital bed density and the CRC stage distribution implies that the impact of bed density may plateau or disappear above certain thresholds. Further research in diverse regions and cancer types is required to identify an appropriate balance between securing medical access and combating poverty.

## AUTHOR CONTRIBUTIONS

**Toshiaki Shibata:** Conceptualization (lead); data curation (lead); formal analysis (lead); investigation (lead); methodology (equal); writing – original draft (lead); writing – review and editing (equal). **Daisuke Shinjo:** Methodology (equal); writing – review and editing (equal). **Kiyohide Fushimi:** Funding acquisition (lead); resources (lead); supervision (lead); writing – review and editing (supporting).

## CONFLICT OF INTEREST STATEMENT

No relevant financial or nonfinancial interests to disclose.

## ETHICS STATEMENT

This study was approved by the Institutional Review Board of the Tokyo Medical and Dental University. Given the anonymized nature of the data, the requirement for informed consent was waived.

## Supporting information


Figures S1–S2.

Table S1.

Table S2.

Table S3.

Table S4.

## Data Availability

DPC data can only be used by licensed facilities, and data access requires approval from the facility. To request the dataset analyzed in this study, please contact the Office of Life Science and Bioethics Research Center: Email infobec@tmd.ac.jp, Phone: +81‐3‐3813‐6111.
